# Sex steroid hormone modulation of neural stem cells: a critical review

**DOI:** 10.1186/s13293-019-0242-x

**Published:** 2019-05-30

**Authors:** Matthew S. Bramble, Neerja Vashist, Eric Vilain

**Affiliations:** Center for Genetic Medicine Research, Children’s Research Institute, Children’s National Medical Center, Washington, D.C., USA

## Abstract

While numerous in vivo experiments have sought to explore the effects of sex chromosome composition and sex steroid hormones on cellular proliferation and differentiation within the mammalian brain, far fewer studies as reviewed here, have explored these factors using a direct in vitro approach. Generally speaking, in vivo studies provide the gold standard to demonstrate applicable findings in regards to the role hormones play in development. However, in the case of neural stem cell (NSC) biology, there remain many unknown factors that likely contribute to observations made within the developed brain, specifically in regions where there are abundant sex steroid hormone receptors. For these reasons, using a NSC in vitro model may provide a more controlled and refined system to explore the direct effects of sex and hormone response, limiting the vast array of other influences on NSCs occurring during development and within adult cellular niches. These specific cellular models may have the ability to greatly improve the mechanistic understanding of changes occurring within the developing brain during the hormonal organization process, in addition to other modifications that may contribute to neuro-psychiatric sex-biased diseases.

## Introduction

Investigating the phenomenon of hormonal organization, or the enduring effects of sex steroid hormone exposure on the brain, became a focal point within the field of neuroendocrinology since the seminal findings of Phoenix et.al was first published in 1959 [1]. While these findings elegantly demonstrated that exposing female fetuses to androgenic compounds resulted in altered adult sexual behavior, the exact mechanisms behind this organization process remain to be fully elucidated. Significant works have built on the hormonal organization theory, and as such, have identified numerous sex-differences in addition to behavior that are set in motion by sex steroid hormone exposures in utero and during the perinatal period [2]. In addition to hormone exposure on the developing brain, it also appears that genetic composition [3, 4] [5, 6] and epigenetic modifications [7–9] significantly contribute to developing adult sexual behavior, sexually dimorphic brain structures, and other sex differences within rodents and humans [10].

While the vast majority of the aforementioned studies have drawn conclusions based on analyses of gross brain tissue, other studies have looked at the direct effects of chromosomal composition and sex steroid influence on specific cells comprising the central nervous system (CNS). Studies have highlighted the effects of testosterone and estrogens on various types of neurons and astrocytes [11–15]; however, few studies to date have explored these effects and the epigenetic consequences of such, on cultured neural stem cells isolated from the embryonic and adult mammalian brains.

Neural stem cells (NSCs) by definition are multipotent populations capable of giving rise to all of the main cell types that comprise the CNS, in addition to having self-renewal capacity [16]—the hallmark of any “stem” cell. There are two general groupings of neural stem cells, those present during early development which will be referred to as embryonic neural stem cells (eNSCs) and those that are maintained during/throughout adulthood (aNSCs). Embryonic neural stem cells are abundant, rapidly dividing, and differentiating during early development, providing sufficient cellular numbers for proper brain formation. These embryonic cells are subject to estrogen and androgen exposures during early development, predominately in utero. Adult NSCs, however, are restricted to specific regions within the mature brain and remain under complex regulatory control within their respective niches [17–19]. aNSCs in theory are exposed to pubertal surges of testosterone and/or estrogen depending on gonadal composition, which remain in abundant circulation for most of adult life. Areas rich in quiescent NSCs during adulthood include the sub-ventricular zone (SVZ) and the sub-granular zone (SGZ) of the dentate gyrus (DG) [20]. Both types of NSCs retain stem properties; however, they appear to have different cellular features and protein expression patterns [16, 20]. This raises the notion that there are intrinsic and extrinsic distinctions to be made between adult NSCs and those present during early brain development, which will be particularly relevant to this review.

Research focusing on neural stem cells and adult neurogenesis has seen an explosion in the past two decades, which has been thoroughly described by Gage and Temple [21]. As noted, despite intense investigation, few studies have sought to explore inherent sex differences and the role that sex steroids have in shaping neural stem cell biology, although studies indicate that such hormones influence adult neurogenesis within the DG [22, 23]. The intent of this review is to highlight the in vitro work that has investigated these aspects in mammalian NSCs, exposing a novel role of sex steroid hormone influence during early brain development and throughout adulthood. Despite being outside the scope of this review, it should be noted that much of our understanding of sex steroid influence on the mammalian brain has been built on studies using the songbird as a research model organism, as reviewed elsewhere [24–27].

### Basal sex-differences in cultured NSCs

**Fig. 1:**
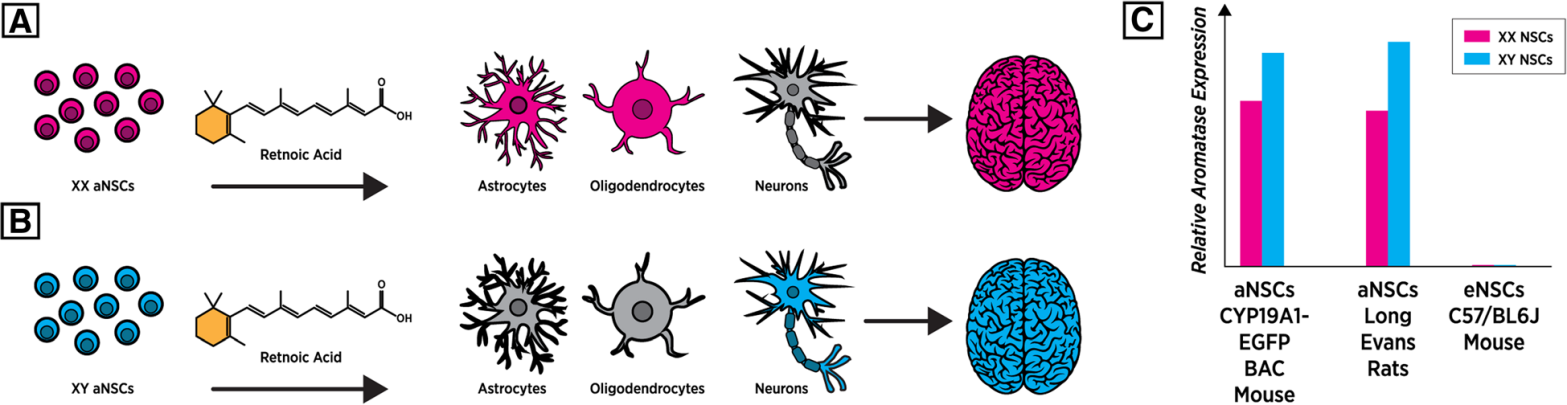
**a**, **b** Upon stimulation with retinoic acid during the differentiation process, XX and XY NSCs show variable differential outcomes, as highlighted post differentiation. Astrocytes and oligodendrocytes are enriched in XX backgrounds post differentiation whereas XY NSCs display a pro-neuronal differentiation pattern. **c** General distributions of aromatase expression found in adult and embryonic NSCs

During analysis of neural stem cells, there have been several studies that have identified inherent basal sex differences between XX and XY NSCs, independent of active androgen or estrogen exposure. One such sex-difference that has been found by several groups is the protein expression level of aromatase, an enzyme responsible for the conversion of testosterone into estradiol, which plays a vital role in hormonal organization of mammalian brains [28, 29]. Using 8–10-week-old adult murine NSCs isolated from the SVZ [30] and NSCs from the SVZ of 3-month-old Long-Evans rats [31], aromatase expression was quantified using both a GFP reporter assay and total protein analysis. Those two independent studies found that aromatase expression in the absence of gonadal hormones displayed a significant male bias with regard to protein expression in adult NSCs. However, our recent study utilizing RNA-sequencing did not identify any expressed aromatase transcripts in either XX or XY murine eNSCs [32] (Fig. 1c). While this discrepancy would typically seem contradictory, our group used NSCs isolated from the telencephalons of E-13.5 C57/B6/J mice, whereas the other two groups used adult-isolated NSCs from both the rat and mouse SVZ. This difference in aromatase expression between adult and embryonic NSCs raises an interesting possibility that the effects of androgenic hormone exposure on this cell type may have markedly different consequences depending on developmental stage. If aromatase is not expressed in murine embryonic NSCs, then during the in utero testosterone surge, the effects on these cells are likely due to direct testosterone signaling. On the other hand, since adult mouse and rat NSCs do express aromatase, there could be downstream effects of pubertal androgenic exposure on these cells. Whether these effects are activational or organizational could be modulated either by direct testosterone action on the androgen receptor (AR) [33] or estrogen signaling, through its various receptors (ERα, ERβ, GPR30) once locally converted into estradiol by aromatase [34].

As earlier mentioned, our group conducted a global transcriptional analysis using RNA-sequencing on eNSCs to determine if there were inherent sex differences with regard to gene expression. To our surprise, we identified 103 transcripts that were differentially expressed between XX and XY murine eNSCs (FDR = 0.10) at a time prior to gonadal-derived hormonal surges [32] The vast majority of these transcriptional sex differences were enriched in pathways predominately involved in cellular replication, possibly indicating that XX and XY eNSCs differently regulate proliferative states. While many of the identified differential transcripts were novel, this was the first study utilizing RNA-seq to probe for sex differences of global gene expression in eNSCs. The identified basal sex differences within eNSCs are quite interesting and provide a unique gene set for further exploration, and it should also be evident that these findings represent a brief snapshot of developmental time, identifying differences that are likely transient. The developing body is highly dynamic and rapidly changing, as are neural stem cells [35]. When assessing early sex differences, it is vital to keep in mind that such findings have spatial and temporal fluctuations and are unlikely to remain static throughout the life of the animal.

Additional research focusing on improving NSC transplantation therapy for various neurological conditions, which still has significant hurdles to overcome [36], identified that both age and sex of NSCs are contributing factors that should be taken into consideration [37]. Using adult NSCs isolated from the SVZ of 3- and 20-month-old Long-Evans rats revealed that upon differentiation in retinoic acid, XY and XX NSCs displayed altered neurogenic and gliogenic potentials, which were both sex and age-dependent. When measuring protein levels, it was determined that neuronal markers such as MAP2 (microtubule-associated protein2), GAP43 (GAP43 growth associated protein 43), and DCX (doublecortin) along with the oligodendrocyte marker CNPase displayed an increased expression pattern in those differentiated NSCs of XY origin [37] (Fig. 1b). The same study found a reverse trend when looking at an astrocyte marker, GFAP (glial fibrillary acidic protein), which showed a very significant enrichment in expression on a XX background (Fig. 1a). The early neuronal maker βIII tubulin did not show sex differences in this study; however, in a later assessment using immunofluorescence, it did appear that βIII tubulin may show sex differences in expression post-differentiation [31]. It was identified that the expression of βIII tubulin, along with those proteins that showed sex differences (GFAP, MAP2, GAP43), were age-dependent and were significantly reduced in differentiated NSCs isolated from 20-month-old rats as compared to those isolated at 3 months of age [37]. The authors concluded that XY NSCs may have inherent potential to differentiate into neuronal linages, while XX NSCs preferentially differentiate down a glia pathway when stimulated with retinoic acid, with cellular age being a large contributing factor. It should be noted, however, that while their differentiation process contained no exogenous gonadal hormones, the XY NSCs from both the 3-month and 20-month-old rats would have been exposed to endogenous testosterone and estrogens prior to isolation. This endogenous exposure may have altered epigenetic programming or specific protein expression, and the conclusions found may not be due to inherent differences based on sex and age, per se, but rather to this possible programming of NSCs induced by endogenous T exposure. This would seem like a possible explanation, as the same group attributed the findings of variances in differentiation potentials to the differences in expression of CYP19 (p-450 aromatase), which was uncovered in their later finding [31].

### In vitro effects of estrogenic compounds on NSCs

**Fig. 2:**
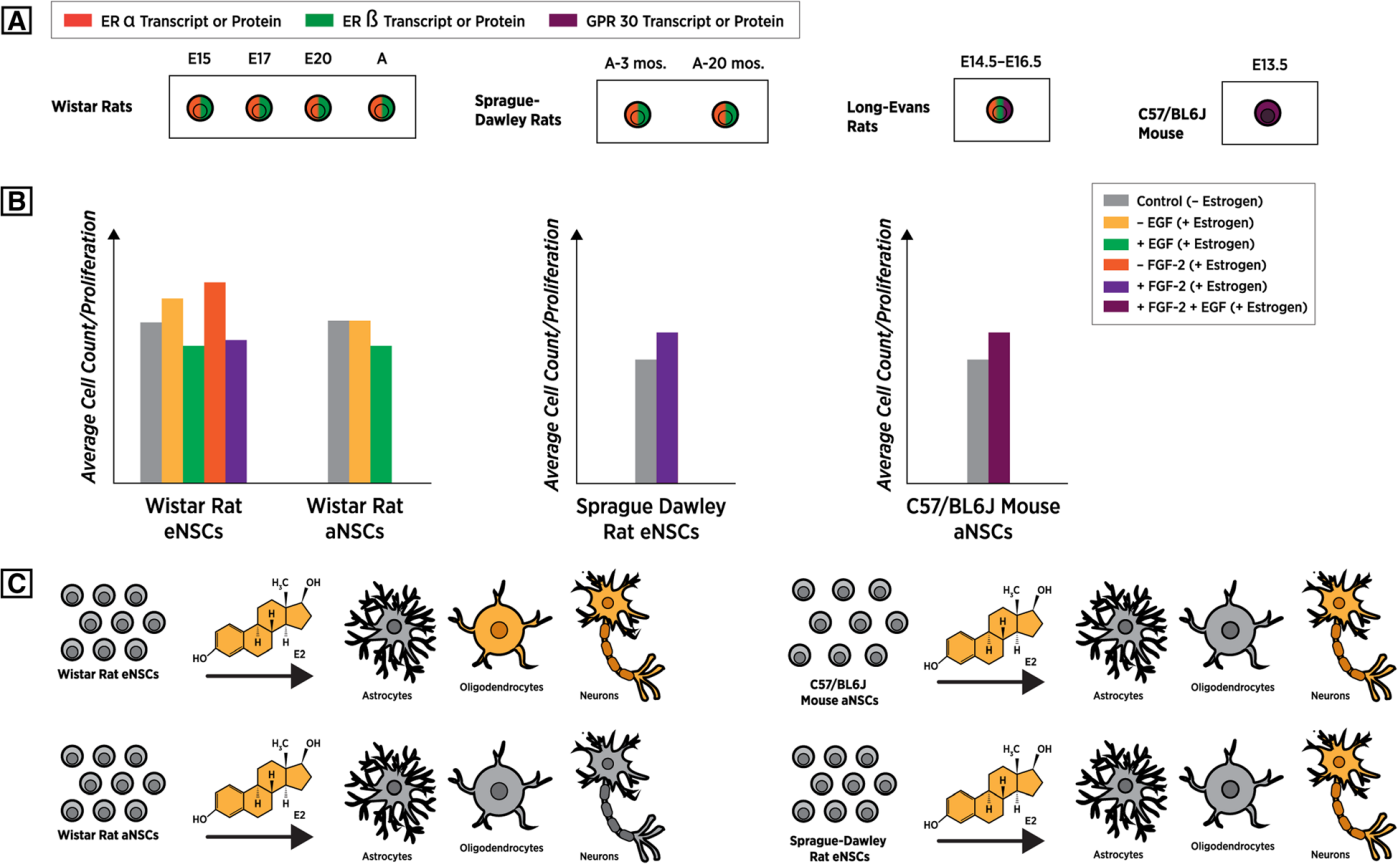
**a** Estrogen receptor expression profiles of NSCs across time and rodent species. **b** The general cellular proliferation affects as a result of estrogen exposure on NSCs isolated from various species of rodents at different time points of development. **c** The effects of estrogen exposure on NSCs during cellular differentiation, highlighting indicates the preferential differential cell outcomes, during or after estrogenic treatment

*Estrogen receptor(s) expression:* In order to determine the effects exerted by estrogens, predominantly 17β-estradiol (E2), on NSCs, it first had to be established that these cell types expressed the appropriate receptors. In one of the first studies assessing the role of E2 on NSC physiology in vitro, it was determined that both embryonic NSCs isolated from E15–E20 Wistar rats and NSCs isolated from the lateral ventricles of adults of the same species, expressed both ERα and ERβ [38] (Fig. 2a) (Table 1). These findings successfully demonstrated that ERα was most expressed in eNSCs during earlier time points of development, when cells were isolated at E15 and E17, and such expression was greatly reduced by E20 and within aNSC populations. The reverse was true for ERβ, which showed increased expression over the E15–E20 developmental period, as well as into adulthood [38]. In studies using aNSCs isolated from the SVZ of 3- and 20-month-old male and female Long-Evans rats, ERα and ERβ were again found to be expressed in both age groups (Fig. 2a) (Table 1), with expression levels being significantly higher in aNSCs isolated at 20 months of age. The same group also claims that ERα and ERβ exhibit different expression patterns based on sex chromosome composition in aNSCs at 3 months of age, but not at the 20-month stage [37]. In a more recent study, the expression of estrogen receptors was again confirmed in eNSCs isolated from E14.5–E16.5 Sprague-Dawley rat embryos. This study however also probed for a newly identified membrane-bound ER receptor, GPR30, and found that in addition to ERα and β, GPR30 protein was also strongly expressed [39] (Fig. 2a) (Table 1). While receptors for estrogens appear to be present in both embryonic and adult NSCs isolated from the rat, the same does not appear to be true for NSCs isolated from embryonic mouse brain tissue. When assaying nuclear hormone receptor expression within NSCs isolated from E-13.5 murine embryos using RT-PCR analysis, it was concluded that both ERα/β were not expressed or were transcriptionally undetectable at that particular time point [40]. Similar findings demonstrating low/no detection of estrogen receptors α/β was also corroborated by our groups complete transcriptomic analysis of E.13.5 mouse NSCs using RNA-Seq [32] (Fig. 2a) (Table 1). While these findings demonstrate that estrogen receptors are at least present in rat NSCs, they also highlight the notion that all NSCs should not be grouped together and the possible response to gonadal hormones such as E2 may be species-specific and variable over both developmental time and in different brain regions.

**Table 1:**
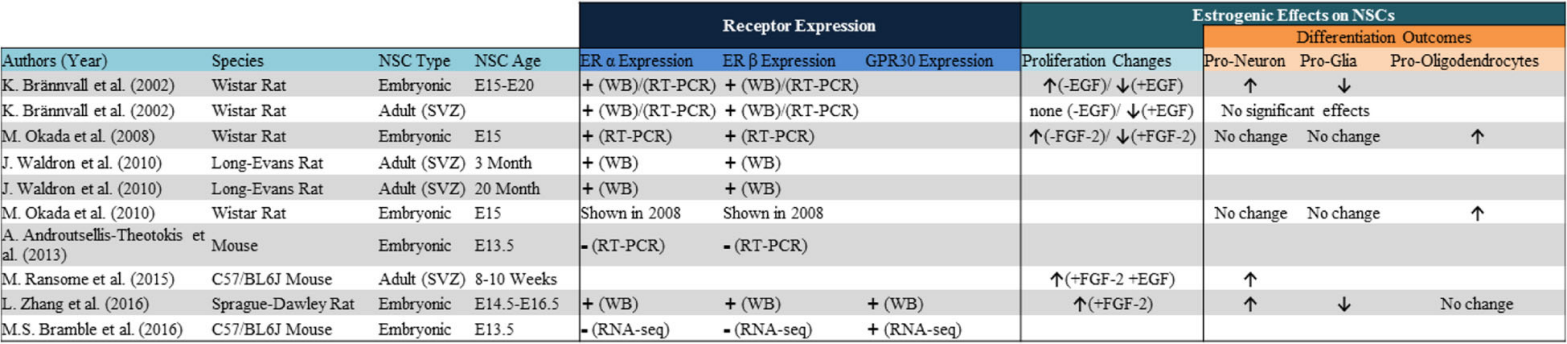
Summary of in vitro studies assessing estrogenic response in neural stem cells

#### The effects of estrogens on NSC cellular proliferation

Studies focusing on the effects of estrogens have predominately investigated how E2 affects cellular proliferation, as well as differentiation potentials of NSC populations. In the Brännvall et.al study, which described the presence of estrogen receptors within these cell types, also assessed the physiological effects of estrogen exposure. Using eNSCs and aNSCs from Wistar rats, it was shown that when 10 nM of 17β-estradiol was introduced to their culture media in the absence of the mitogen EGF (epidermal growth factor), there was a significant (7%) proliferation increase of eNSCs, as measured by BrdU-positive cells [38] (Fig. 2b) (Table 1). This effect was not seen in their aNSC population, or when the ER antagonist ICI-182,780 was present. When the same assay was conducted in the presence of EGF, there was actually decreased proliferation of both eNSCs and aNSCs (Fig. 2b) (Table 1). This decrease in proliferation in the presence of EGF was attributed to the upregulation of the cyclin-dependent kinase (CDK) P21, as it was shown that protein expression of this cell-cycle regulator was significantly increased in the presence of E2. The authors note that while E2 can moderately increase proliferation of embryonic derived NSCs, this effect is greatly dependent on other growth factors, as in their case, EGF. While a 2008 study investigating the effects of E2 on rat eNSCs was unable to replicate these initial findings, using a modified media composition still demonstrated that estradiol alone was capable of increasing proliferation of eNSCs isolated from the telencephalons of E15 Wistar rat embryos [41]. The same conclusions were also drawn from both a 2010 [42] and 2016 study; however, it was also determined that increased doses of E2 (50 nM) actually had the opposite effect, resulting in a reduced proliferative state [39] (Table 1).

From these independent studies, it can be concluded that while E2 can significantly increase proliferation of rat eNSCs through ER action (Fig. 2b), these outcomes are greatly dependent on both the dose of estrogen and the site of NSC isolation. To our knowledge, only one study has assessed the effects of estradiol exposure on adult-derived mouse NSCs and also found that E2 significantly increased cellular proliferation, using a Ki67 proliferation marker, regardless of chromosomal sex [30] (Fig. 2b) (Table 1). This work did not show estrogen receptor expression per se and as previously stated such transcripts have not been identified in murine eNSCs by other groups. However, given their results, it can be inferred that perhaps estrogen receptor expression, while not present at the embryonic stages, becomes expressed by aNSCs in the murine SVZ and stimulation with E2 elicits proliferative effects in the adult mouse also.

#### The effects of estrogens on NSC differentiation

In addition to proliferation, many of the studies assessing those effects also investigated the role that E2 plays during the differentiation process. When eNSCs isolated from the rat were treated with 10 nM of E2 while undergoing a 4-day differentiation, it was found that there was a significant increase in the ratio of βIII tubulin-positive neurons over GFAP-expressing cells (Fig. 2c) (Table 1). The same outcome was not seen when aNSCs were treated with E2 during differentiation, indicating again that embryonic and adult cells may have completely different responses to estrogens (Fig. 2c) This pro-neuron effect was attenuated when the ER antagonist ICI-182,780 was added to the media, demonstrating that these observations during differentiation were modulated in part by ERα/β signaling [38]. Recent publications came to similar conclusions and determined that 10 nM of E2 stimulated differentiation to proceed down a neural lineage as determined by the ratio of Tuj-1 (βIII tubulin marker) to GFAP signal (Fig. 2c) (Table 1). These findings again demonstrated that 10 nM of E2 seems to be the most optimal dose for stimulating this differentiation outcome, as 1, 20, and 50 nM concentrations of E2 did not result in altered neuron/glia ratios [39]. There also appears to be no sex differences in the effects of E2 on NSC differentiation in cultured adult murine cells, where both XX and XY lines showed increased neuronal staining patterns when allowed to differentiate in the presence of 10 nM of E2 [30].

Two studies conducted by Okada et.al using embryonic-derived rat NSCs were unable to replicate the findings showing preferential neuron differentiation upon stimulation with E2 [41, 42] (Table 1). While this group did not observe increased neuronal potential, they did however determine that E2 increased differentiation of both CNPase-positive oligodendrocytes and NG-2-positive oligodendrocyte precursor cells (Fig. 2c). The authors concluded (but did not demonstrate) that this effect was not a result of classical ER signaling, but rather membrane ER signaling, because when NSCs were pre-treated with ICI-182,780, the ratios of oligodendrocytes and precursor cells were still increased during differentiation in the presence of E2 [41, 42]. It should also be noted, that while all studies are not in agreement with regard to pro-neuronal outcomes when NSCs are stimulated with E2, there were also technical differences between experiments that may have played a factor. The studies that identified E2 as a pro-neuronal stimulant [38, 39] grew their NSCs in the presence of EGF, whereas the studies that were unable to replicate these findings and saw pro-oligodendrocyte differentiation [41, 42] grew their NSCs with the mitogen FGF-2 (fibroblast growth factor 2) (Table 1). It has been established that murine embryonic NSCs express FGF and EGF receptors in a temporal and spatial manner, and as such, respond to these mitogens in different ways [43], highlighting the importance of what otherwise may seem like a minor technical difference between studies.

### Conclusions of the effects of estrogens on NSCs

Prior to these in vitro applications using NSCs, groups have demonstrated the effects of sex and estrogens on eliciting both cellular proliferation and increased neurogenesis within the adult rodent brain, predominantly within the DG region of the hippocampus [23]. In vivo assessments have shown that cycling female adult SD rats display altered states of cellular proliferation within the DG, in accordance with various time points during the estrous cycle. Increases of cell proliferation were observed in the DG during proestrus, when circulating levels of estrogens were at their peak and subsequently declined during estrus, when estrogens were at lower levels [44]. However, if cellular proliferation is assessed prior to the onset of the estrous cycle, it appears that sex differences exist, showing a male bias increase in cellular proliferation within the same brain region, as measured by BrdU labeling [45, 46]. These reviewed in vitro applications (Table 1) have also drawn similar conclusions, in at least cells isolated from embryonic stage SD rats, where E2 alone can elicit increased cellular proliferation. Interestingly, these proliferation increases were not seen in adult-isolated NSCs from the SVZ, which may indicate that while estrogens elicit proliferation in the DG, the same events may not occur within the SVZ. Additionally, the same strain of rat was not used for all studies, and data indicates that cellular proliferation within the DG is variable between strains [45], a factor that should be taken into consideration. In vitro evidence also supports that both doses of estrogen as well as other modulators such as EGF and FGF contribute to the observed effects, as E2 in the presence of such mitogens actually causes a reduction in proliferation. Similar outcomes have also been observed in proliferating cells within the DG, where E2 dosage was a contributing factor, as well as the presence of other variables, such as progesterone, which essentially reverses the increased proliferation effects of estrogen [47].

These reviewed in vitro studies have shown that NSCs isolated from the adult mouse SVZ show increased proliferation in the presence of E2, but in vivo approaches have observed the opposite, indicating that estrogens decrease proliferation within the SVZ [48]. When focusing on cellular propagation within the mouse DG, It was found neither sex nor estrogens stimulated adult neurogenesis or increased cellular proliferation in the C57/BL6 mouse strain [49]. This again shows that there are important distinctions to be made between the site of NSC isolation and the mammalian species from which they arose, grouping NSCs of different ages or from different regions not recommended.

Based on these few studies, it can be concluded that estrogen exposure on NSCs isolated from embryonic regions elicits altered differentiation potentials, increasing the ratios of neurons over GFAP expressing glia cell types. This effect appears to be restricted to embryonic stages, as estrogen stimulation on aNSCs in vitro did not have the same outcome, an effect also observed within the adult DG post-estrogen treatment [50]. However, as noted, not all groups observed this difference in neuron/glia outcome; those that did not detect a pro-neuronal outcome as a result of estrogenic stimulation did detect an increase in oligodendrocyte differentiation.

Generally speaking, both the in vivo studies and in vivo studies have drawn similar conclusions when assessing the effects of estrogen stimulation on NSCs in culture or progenitor cells within the adult rodent brain. Despite a growing body of evidence now indicating that estrogen stimulation can have dramatic epigenetic effects within the brain [8, 51], no study to date has directly assessed these effects on neural stem cells or progenitor cells within the brain. Considering that these cells respond to estrogens, these NSC in vitro models will prove to be extremely useful for the study of epigenetic changes ensuing post-estrogen stimulation. These methods will limit the numerous variables occurring within the mammalian brain and allow for a more direct assessment of estrogen influence on epigenetic alterations such as DNA methylation, histone modifications, and the ensuing gene expression outcomes.

### In vitro effects of androgenic compounds on NSCs

#### Androgen receptor expression

While several in vitro approaches have provided evidence supporting the role of estrogens in proliferation and differentiation of cultured NSCs, few studies have addressed how such cells respond to androgenic compounds, despite known androgen response in other types of stem cells [52]. Brännvall et.al demonstrated that both embryonic and adult rat NSCs derived from the SVZ of Wistar rats expressed the androgen receptor (AR). Using RT-PCR and western blots, the group showed that AR expression was most abundant in embryonic NSCs as compared to aNSCs, with peaks of expression occurring between gestational days E15 to E17 [53] (Table 2). Androgen receptor also appears to be expressed in both XX and XY aNSCs isolated from the adult mouse SVZ and does not appear to show a sex difference in protein expression. Unlike the results demonstrating low/no estrogen receptor gene expression in mouse eNSCs, this same study [40], along with our groups RNA-seq findings, did identify that AR was transcriptionally detectable and non-dimorphic in both male and female murine NSCs isolated from gestational day E13.5 embryos [32] (Table 2). While limited, these independent studies identified that both embryonic and adult NSCs isolated from the rat and mouse express AR and therefore should be responsive to various types of androgen exposure.

**Table 2:**
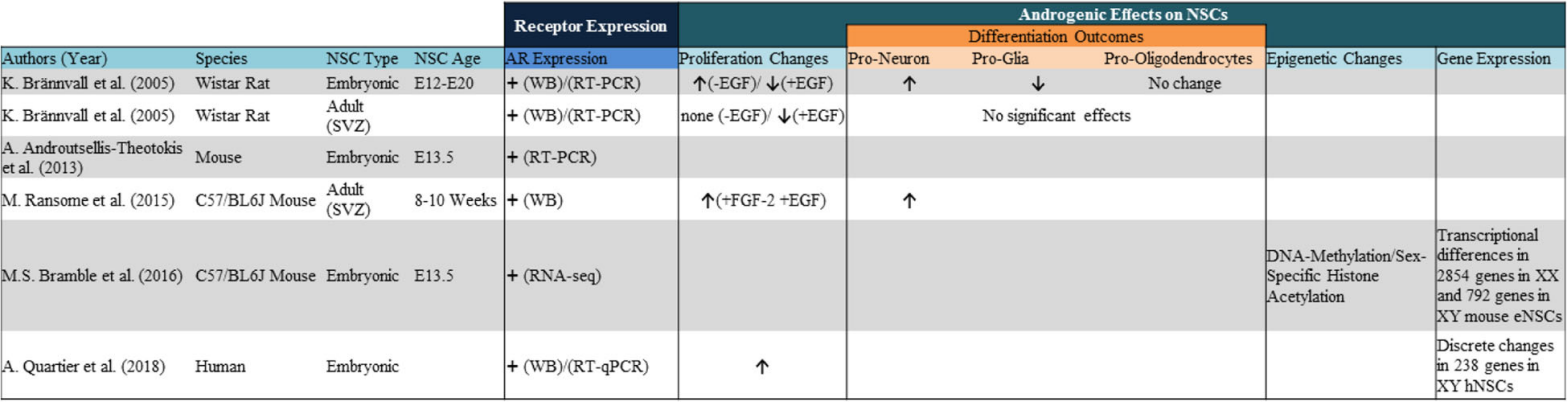
Summary of in vitro studies assessing androgenic effects on neural stem cells

#### The effects of androgens on NSC cellular proliferation

**Fig. 3:**
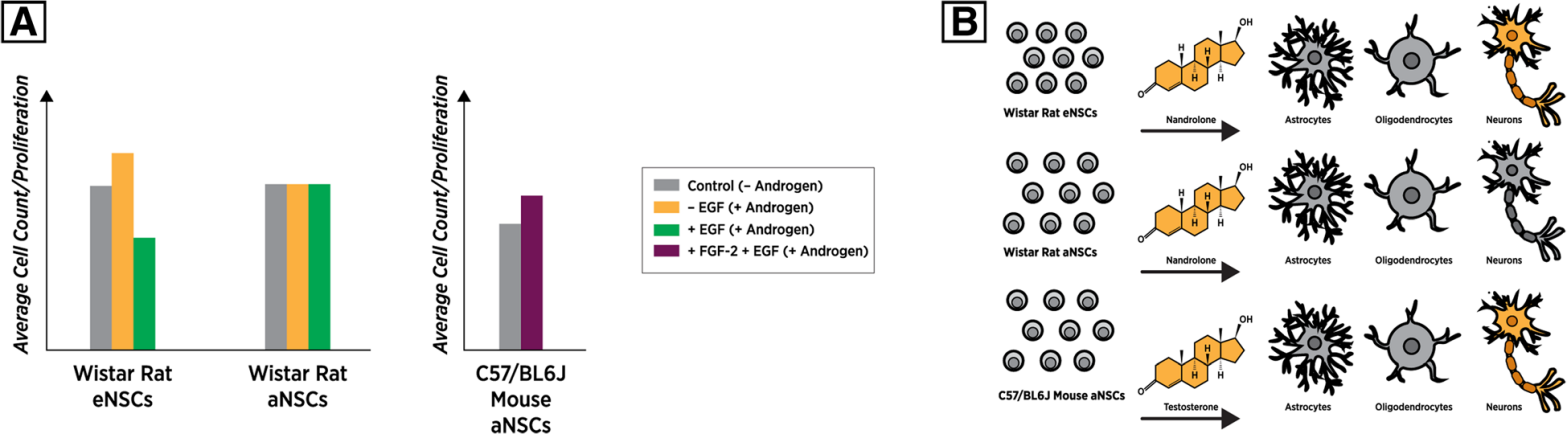
**a** General trends for cellular proliferation outcomes when NSCs are treated with androgens and various growth factors. **b** General observed trends for final cellular outcomes when NSCs are treated with androgenic stimulation during differentiation, highlighting indicates the cell types that were preferentially favored post-treatment with androgenic compounds

When both embryonic and rat aNSCs were cultured in the presence of the androgen nandrolone (19-nortestosterone), along with the mitogen EGF, proliferation was decreased by 30% in eNSCs and 20% in aNSCs (Fig. 3a) (Table 2). However, if cultured in the absence of EGF with nandrolone, there was roughly a 7% increase in proliferation of eNSCs, but this effect was not observed in adult female-derived NSC populations (Fig. 3c). It was determined that these alterations in proliferation were modulated in part by androgen receptor action, as treatment with the AR antagonist flutamide quelled some, but not all of nandrolone’s effects [53] (Fig. 3a) (Table 2). Again, the authors probed for differences in various cell-cycle regulators that may be responsible for proliferation changes as a result of androgen exposure and determined that unlike their findings using E2 [38], CDK P-21 was not upregulated as a result. As concluded by the group, despite similar proliferation differences in response to E2 and nandrolone in the presence of EGF, the pathways responsible for such outcomes appear to be modulated by different mechanisms of action.

After measuring the effects of various concentration of testosterone (T) on the proliferation of murine aNSCs, Ransome et.al demonstrated that while 1 nM T did not produce proliferative differences, 10 and 50 nM of T significantly increased proliferation of XX and XY aNSCs, even in the presence of both EGF and FGF-2 (Fig. 3a) (Table 2). The increased proliferation as a result of T exposure was determined to result from Erk phosphorylation induced by MEK-1, as sex hormone influences were ablated in the presence of U0126, a MEK1 phosphorylation inhibitor [30]. This particular study also found interesting sex differences in how testosterone elicited these responses in aNSCs. It was determined that while T induced proliferation in both sexes, XX aNSC growth was inhibited by flutamide, but XY aNSCs were still capable of increased proliferation even when AR was actively subdued [30]. This demonstrates that the conversion of T into E2 via aromatase can also modulate cellular proliferation in XY but not XX cells, highlighting the fact that male and female adult NSCs have differential response mechanisms when exposed to specific hormones. These two studies indicate that androgenic compounds can have significant effects on NSC proliferation, albeit in opposite directions, perhaps again revealing that NSCs isolated from different species respond differently to stimulations by sex-steroid hormones. Cellular proliferation also appears to be increased in human-derived XY neural stem cells (hNSCs), when treated with the more potent androgen, dihydrotestosterone (DHT). The mechanism of action to increase proliferation in hNSCs is AR-dependent, and it was demonstrated that when siRNA was used to ablate AR signaling, proliferation differences were not observed in the presence of DHT [54]. It remains unclear if there are sex differences in this hNSC response, as this particular study utilized only the XY line for proliferation experimentation (Table 2).

#### The effects of androgens on NSC differentiation

The groups that have assessed proliferative effects of testosterone on NSCs also addressed the role of androgens on differential outcomes, post-NSC differentiation. If nandrolone was present during a 5-day differentiation process of rat eNSCs, the proportion of βIII tubulin-expressing neurons was higher than GFAP-expressing cells, an effect not seen in adult NSCs (Fig. 3b) (Table 2). This effect was ablated when flutamide was present, indicating that this effect was modulated by androgens binding AR [53]. The Ransome et.al study also showed that the presence of 10 nM T during a 2-day murine aNSC differentiation resulted in an increase of βIII tubulin-positive cells, in both XX and XY lines [30] (Fig. 3b). Like E2, it appears that androgens also have a pro-neuronal influence during differentiation of embryonic NSCs from the rat and adult NSCs from the mouse. Due to lack of experimental evidence, it remains unclear if the same differentiation outcome occurs in embryonically derived murine NSCs.

#### The effects of androgen exposure on the transcriptome and epigenome of NSCs

Findings published by our group aimed to explain many of the unanswered questions regarding the development of the sex differences in the brain and the role of androgens, mainly testosterone on such events. To model the organization of the prenatal brain, we assessed the global transcriptional and epigenetic changes that occur as a result of testosterone propionate (TP) exposure on murine eNSCs. We determined that 20 nM of TP resulted in 2854 transcriptional differences on a XX background, and 792 gene transcript expression differences in XY eNSCs, using a false discovery rate of 10% (FDR = 0.10) [32]. While TP had more robust effects on a XX genetic background, 600 of these differentially expressed transcripts were mutually shared between the XX and XY cells, indicating that there are both sex-chromosome independent as well as dependent effects of TP exposure on eNSCs gene transcription (Table 2). Interestingly, it was recently shown that the more potent form of testosterone, dihydrotestosterone (DHT), exposure on human-derived NSCs also elicited differences in gene expression. Many of the observed changes in transcription post-DHT exposure on a human XY background were enriched in genes that have been associated with autism spectrum disorders [54]. These changes in gene expression were ablated when AR was inhibited, indicating that the observations were due to direct androgen signaling. In addition to detecting gene expression differences as a result of androgen exposure, TP significantly reduced global levels of 5-methylcytosine during active exposure, an epigenetic modification that was apparently transmissible to daughter cells in the absence of androgen. Another epigenetic modification that showed androgen sensitivity was acetylation levels of histone tails, which were found to be modified in a sex-dependent manner [32] (Table 2). Although additional studies will be necessary to solidify these findings, these data indicate that androgens not only have the capacity to greatly influence gene transcription, but also various epigenetic modifications within murine eNSCs.

## Conclusions

Unlike studies involving estrogen stimulation on NSCs, very few studies have demonstrated the effects of androgens on the same cells. The limited evidence supports that androgens can induce proliferation of embryonic rat NSCs in the absence of growth mitogens; however, if present in culture, androgen stimulation reduces proliferation (Fig. 3a). This effect was not observed in adult-isolated NSCs from the rat SVZ, indicating that androgen stimulation on embryonic populations are markedly different than when assayed using adult NSCs (Fig. 3a) In vivo studies have also found that that androgenic stimulation does not increase proliferation in the DG of adult rats nor does there appear to be AR expression within that region [55, 56]. This indicates that AR is expressed and responsive to androgens within adult cells isolated from the SVZ, however, not from other neurogenic niches such as the DG. The same also appears to be true for NSCs isolated from the SVZ from the mouse, where testosterone exposure can increase proliferation in both XX and XY NSCs.

Differentiation outcomes appear similar to those found with estrogen stimulation, showing that androgenic treatment can increase the ratio of neurons to glia during cultured differentiation of embryonic rat NSCs and adult murine NSCs from the SVZ (Fig. 3b). Despite in vivo studies showing that androgen did not increase proliferation in the DG, prolonged androgen exposure did increase neurogenesis by enhancing the survival of adult-born neurons within this region [55]. Our group’s findings indicating that testosterone exposure can significantly reduce DNA methylation and alter histone tail acetylation within murine eNSCs indicate that androgenic exposure also has significant consequences on the NSC epigenome.

While in vivo findings have shown that testosterone can alter DNA methylation [7] in gross brain regions, our group showed that these effects are also observed in cells at early time points of brain development. This raises the possibility that early androgen exposure may elicit significant developmental, as well as behavioral, outcomes by reprogramming the epigenome of NSCs. These outcomes may contribute significantly to normal male behavioral development, as XY rodents with inactive AR (Tfm model) display reduced male-typical aggressive and sexual behaviors in adulthood, despite functional estrogen receptors [57]. Though still in the very early stages, it is likely that deeper investigations into the roles of estrogens and androgens on neural stem cells will significantly improve our knowledge of sex steroid hormone response on the embryonic and adult brains. These future investigations will likely lead to a more complete and comprehensive understanding of the early mechanisms behind hormonal organization of the mammalian brain and some of the factors that lead to sex differences in neuro-psychiatric disease.

## Data Availability

Not applicable

## Abbreviations

aNSCs: Adult neural stem cells
AR: Androgen receptor
CNS: Central nervous system
DCX: Doublecortin
DG: Dentate gyrus
DHT: Dihydrotestosterone
E2: 17β-estradiol
EGF: Epidermal growth factor
eNSCs: Embryonic neural stem cells
ERα: Estrogen receptor alpha
ERβ: Estrogen receptor beta
FGF: Fibroblast growth factor
GAP43: GAP43 growth associated protein 43
GFAP: Glial fibrillary acidic protein
MAP2: Microtubule-associated protein2
NSCs: Neural stem cells
SGZ: Sub-granular zone
SVZ: Sub-ventricular zone
T: Testosterone
TP: Testosterone propionate
